# Feasibility of the AusMed Diet Program: Translating the Mediterranean Diet for Older Australians

**DOI:** 10.3390/nu12041044

**Published:** 2020-04-10

**Authors:** Karly Zacharia, Amanda J. Patterson, Coralie English, Lesley MacDonald-Wicks

**Affiliations:** 1 School of Health Sciences, Faculty of Health, University of Newcastle, University Drive, Callaghan, NSW 2308, Australia; amanda.patterson@newcastle.edu.au (A.J.P.); coralie.english@newcastle.edu.au (C.E.); lesley.wicks@newcastle.edu.au (L.M.-W.); 2 Priority Research Centre for Stroke and Brain Injury, University of Newcastle, University Drive, Callaghan, NSW 2308, Australia; 3 Priority Research Centre for Physical Activity and Nutrition, University of Newcastle, University Drive, Callaghan, NSW 2308, Australia

**Keywords:** dietary intervention, prevention, Mediterranean diet, dietary behaviour change, intervention development, intervention evaluation, chronic disease

## Abstract

The Mediterranean diet pattern (MEDI) is associated with a lower risk of chronic conditions related to ageing. Adherence research mostly comes from Mediterranean countries with high cultural acceptability. This study examines the feasibility of a MEDI intervention designed specifically for older Australians (AusMed). Phase 1 involved a consumer research group (*n* = 17) presentation of program materials with surveys after each section. In-depth individual semi-structured interviews (*n* = 6) were then conducted. All participants reported increased knowledge and confidence in adherence to the MEDI, with the majority preferring a booklet format (70%) and group delivery (58%). Three themes emerged from interviews—1. barriers (complexity, perceived cost and food preferences), 2. additional support and 3. individualisation of materials. Program materials were modified accordingly. Phase 2 was a 2-week trial of the modified program (*n* = 15). Participants received a group counselling session, program manual and food hamper. Adherence to the MEDI was measured by the Mediterranean Diet Score (MDS). All participants increased their adherence after the 2-week trial, from a mean score of 5.4 ± 2.4 (low adherence) to a mean score of 9.6 ± 2.0 (moderate to high adherence). All found that text message support helped achieve their goals and were confident to continue the dietary change.

## 1. Introduction

As the world’s population continues to rapidly age [1], we see an increase in the incidence of age-related chronic disease [2]. Australia’s population is no exception. Over 3.5 million people (15.1%) are aged >65 years [1] and 80% have one or more chronic conditions [3]. People are living longer but not necessarily healthier lives. Chronic disease can be modified by a change in diet [4], but dietary change is difficult and requires support. Developing interventions specific to an ageing population is needed to improve health outcomes. 

One of the most studied dietary patterns is the Mediterranean diet pattern (MEDI). The MEDI includes a high intake of fruits and vegetables, nuts, wholegrains and legumes. Intake of commercial sweets, red and processed meats is limited, and fish, poultry and dairy foods are eaten in moderation. Olive oil is the main source of fat and wine is recommended in moderation with meals [5]. Observational studies have shown that the MEDI is associated with a lower of risk chronic disease [6]. A recent Umbrella Review (*n* > 12,800,000) compared MEDI adherence and multiple health outcomes. It found that greater adherence correlated with a positive effect on overall mortality (0.93, CI 95% 0.65, 1.33), cardiovascular disease (CVD) (0.62, CI 95%, 0.45, 0.86), coronary heart disease (0.56, CI 95%, 0.20, 1.61), myocardial infarction (MI) (0.60, CI 95%0.44, 0.82), stroke (0.64, CI 95%, 0.47, 0.86), Alzheimer’s disease (0.60, CI 95%, 0.48, 0.77) and dementia (0.69, CI 95%, 0.57, 0.84) [6].

We also know that the benefits of the MEDI have been translated to non-Mediterranean countries [7] and that several studies have shown that adherence is possible in an Australian setting. For example, a 12 week trial (the “SMILES” trial; Supporting the Modification of lifestyle In Lowered Emotional States) modified the MEDI to make it suitable for Australian products and seasonal produce and reported high adherence (as measured by a modified MDS) by adults (mean age = 40.3 years) with major depression [8]. This research included education and behaviour change strategies as part of its protocol [9,10]. A 6 month Australian RCT (the Medley Study) [11] showed that older Australians could adhere to a modified MEDI but their adherence scores were lower than those from the European Prospective Investigation into Cancer and Nutrition (EPIC) and Prevención con Dieta Mediterránea (PREDIMED) studies [12,13]. The study protocol contained no behaviour change strategies [11], which may account for lower adherence. In addition, a 12 month follow up to this study showed a further drop in adherence (MDS: 4 months = 9.6 ± 0.2, 18 months = 7.9 ± 0.3) and the study authors concluded that further dietetic support may be needed to maintain adherence [14]. 

We have developed a dietary intervention to support older Australians to adhere to the principles of the MEDI (AusMed) while incorporating the Australian Guide to Healthy Eating recommendations (AGHE) [15]. The intervention includes a package of materials: a 2 week meal plan with recipes modified to be familiar to this population, shopping lists, education and behaviour change support materials. This package aims to make an AusMed diet more accessible and more appropriate for older Australians to increase the rate of adherence to the MEDI and therefore improve overall health. The aim of this research is to test the feasibility of this dietary intervention and to assess perceived barriers and enablers in order to support older Australians to adhere to an AusMed diet pattern. We hypothesise that tailoring a MEDI intervention to be specific to the needs of older Australians may leads to an increase in adherence.

## 2. Materials and Methods

**Figure 1:**
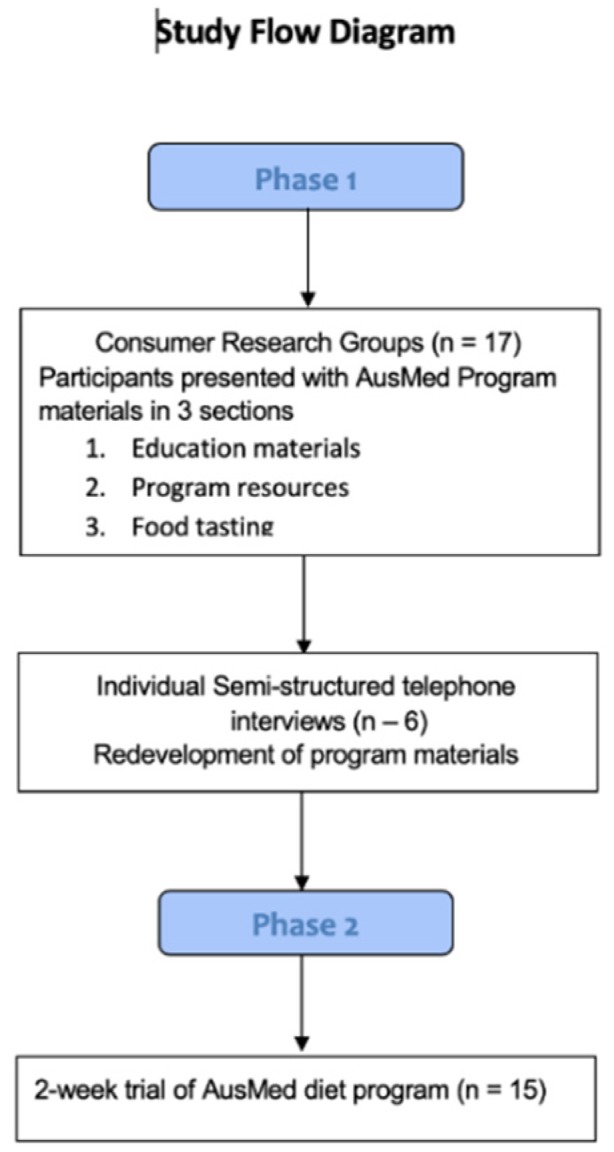
Flow chart of the study processes.

This study consisted of 2 phases, the first was a process evaluation to assess the acceptability of the AusMed diet program resources and materials in a population of older Australians. The second phase was a 2-week feasibility trial of the diet program itself. Figure 1 displays an overview of the study processes. All participants provided written, informed consent prior to participation in each phase. This study was approved by the University of Newcastle Human Research Ethics Committee, (#H-2018-0217). Phase 1 was conducted in September 2018 and Phase 2 took place between February and March 2019.

### 2.1. Phase 1 Process Evaluation

The process evaluation of the AusMed diet program materials used a mixed-methods approach. Data were collected from a sample of older Australians (>55 years) to assess the acceptability of the program materials. Quantitative data were collected via surveys conducted after presentation of the AusMed diet program materials at a consumer research group session and qualitative data were collected from follow-up semi-structured telephone interviews.

#### 2.1.1. Phase 1 Participants

Seventeen participants were recruited—12 from a retirement community using their closed resident email and Facebook™ groups and 5 using convenience sampling. Inclusion criteria were that participants be over the age of 55 years and able to attend the University of Newcastle Food Science Laboratory for a 2–3 h consumer research group session. As there was a food sampling requirement within the session, participants were excluded if they had a diagnosed food allergy to nuts, eggs or seafood, had a diagnosed/reported food intolerance to gluten or dairy OR reported any other dietary restriction such as a gluten free or dairy free diets.

#### 2.1.2. Phase 1 Study Design

Participants were invited to attend consumer research sessions at the University of Newcastle Food Science laboratory. Background data were collected via a paper-based survey on arrival which included demographic information as well as diet/food preparation-specific information and confidence level in food preparation. Participants were also asked to indicate whether they would be willing to complete a follow-up individual semi-structured interview. Participants attended a single session lasting 2–3 h, where a trained moderator presented the AusMed diet program materials divided into three sections:

1. The AusMed education materials—background, diet–disease relationship, staple foods, plate ration and food pyramid.
2. The AusMed diet program itself—menu plan, recipes, shopping lists, troubleshooting the menu plan.
3. Foods typical to the Mediterranean diet—cooking demonstration and sample tasting of three recipes from the program.

Evaluation survey data were collected from participants after each presentation section. Likert scales were utilised to assess participants’ opinion of the acceptability of the materials and their confidence in their capability of following the AusMed diet program. Participants indicated their agreement with five statements with a score ranging from one, indicating strong agreement, to five, indicating strong disagreement. Participants could also provide written comment at the end of each survey section.

Following consumer research group sessions, those willing to participate in the semi-structured telephone interviews were given an information statement which included advice that member checking (provision of the qualitative data collected via their individual telephone interview for respondent feedback) would not be feasible due to time constraints of the project but that they could redact or alter any part of their interview. Informed consent was then provided, and interview times were scheduled.

Individual semi-structured telephone interviews lasting 12 to 21 min were conducted with (*n* = 6) participants to provide in-depth qualitative data on the acceptability of the AusMed materials. Participants were selected in order of response to the interview request and were from across the 3 consumer research groups. Interviews were recorded and transcribed verbatim via Temi (an iOS application) using a proprietary algorithm and voice-recognition software. No human had access to the recorded files. The site is secured via TLS 1.2 data encryption and the files are deleted permanently from the server post delivery of the transcription Word document to the researchers via email. The transcription document was checked against notes and the memory of the interviewer for accuracy.

Interviews were shaped by a topic guide including nine questions with prompts. Interview questions were open ended and included topics such as the participant’s opinion of the physical materials (ease of reading, font size, colour), knowledge of the foods presented in the program and their likelihood of consumption, self-efficacy in terms of their capability to access foods, shop and prepare dishes, barriers or enablers to the participants perception of their ability to change dietary patterns and any suggested improvements to the program or materials. They were asked to indicate whether the program materials had encouraged them to change their diet. Interviews were repeated until data saturation was reached and no new themes or key points emerged.

#### 2.1.3. Phase 1 Outcome Measures

Data from the three evaluation surveys of the consumer research group presentations were collated and quantified using Excel and analysed using descriptive statistics. This information was then used to refine the structure, questions, probes and prompts of the telephone interviews. 

The transcribed data from the semi-structured telephone interviews and the individual participant comments from the surveys were analysed using standard 6 phase qualitative thematic analysis to extract common themes utilising NVivo software: 1. familiarisation of data; 2. coding; 3. extracting themes; 4. review of themes; 5. defining the themes; 6. writing—contextualising themes in relation to existing literature [16]. This process was iterative and repeated sweeps of the data were conducted to ensure that any meaning derived was supported. A second researcher reviewed the thematic analysis to confirm the findings. 

### 2.2. Phase 2 Pilot Trial of AusMed Diet Program

As a result of the information gathered from Phase 1 of the study, the AusMed diet program was modified to include weekly menu plan and shopping list templates and an education session to enable participants to individually tailor the program. A text message support system was created using the Behaviour Change Wheel and COM-B model (which utilises a person’s Capability, Opportunity and Motivation to elicit a change in behaviour) in order to design the message strategy [17] and provisioning for the staple foods hamper to the AusMed diet program was made. 

#### 2.2.1. Phase 2 Participants

Participants in Phase 1 of the study were recruited for a 2-week trial of the AusMed diet program. Of the Phase 1 participants (*n* = 17), 13 participants provided written, informed consent to trial and 4 participants dropped out due to travel commitments. A further 2 participants were recruited via word of mouth, giving a total of *n* = 15 participants for the trial. 

#### 2.2.2. Phase 2 Study Design

Phase 2 was a trial of the AusMed diet program. Participants attended a single face-to-face group session of 2–3 h where a trained moderator delivered the AusMed diet program trial in 3 parts; 1. education materials, 2. AusMed diet program and how to use templates to tailor the diet individually and 3. individual goal setting. Participants received instructions on the timeframe for completion of the 2 week trial of the AusMed diet program and completed a pre-trial validated 14-point Mediterranean Diet Score [18]. Participants were provided with a sample 2-week menu plan and shopping list, an associated recipe book and a staple foods hamper to the AusMed diet program including olive oil, nuts, wholegrains, spices, tinned tomatoes and legumes. Other resources included education material, troubleshooting advice for recipe modification, recommended daily/weekly servings of core food groups and templates to create their own meal plan and shopping list.

For the following 14 days, participants followed the diet program and received twice weekly text messages designed using the COM-B model [19], with a goal to support behaviour change and increase adherence (supplementary Table S1). Participants were sent post intervention emails with the Mediterranean Diet Score survey and evaluation surveys to assess the food and text message components of the program. 

#### 2.2.3. Phase 2 Outcome Measures

Phase 2 outcomes measures were a change in adherence to a Mediterranean dietary pattern. We calculated the mean and standard deviation for the change in overall diet score and each numerical variable of the validated 14-point Mediterranean Diet Score and the percentage for the two categorical variables, and then tested for significance using paired t-tests and Fisher’s exact test (*p* < 0.05 = significant). Data from the food and text message surveys were collated and quantified using excel and analysed using descriptive statistics.

## 3. Results

### 3.1. Participant Characteristics

In total, 17 participants attended one of the three consumer research groups and 15 participants completed the trial of the AusMed diet program. Volunteers were older Australians (mean age 71.2 ± 4.2 years, females = 70.7 ± 4.2 years, male = 72.8 ± 4.2 years) who were retired (94%) or participated in unpaid voluntary work (6%) (Table 1). 

The majority of participants reported that they were either confident or very confident in all aspects of food preparation when surveyed prior to the research group presentation. All participants reported feeling confident or very confident in all aspects of accessing foods (transportation, list making and shopping) and recipe reading. In terms of preparing food for consumption, 18% of participants reported that they were neutral or not confident in cooking from a recipe. However, 94% felt confident in preparing meals for the day (Supplementary Figure S1). 

### 3.2. Phase 1 Quantitative Analysis

#### 3.2.1. Phase 1 Nutrition Education Materials Survey

**Figure 2:**
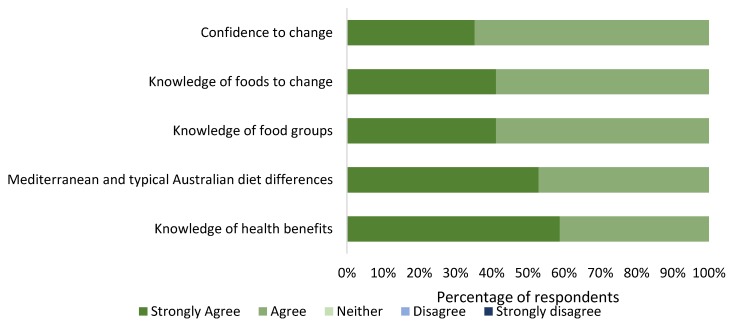
Consumer research group participant (*n* = 17) opinions on the applicability and effectiveness of education materials and their ability to implement the AusMed diet program.

All participants (100%) indicated that their knowledge of the Mediterranean diet, its health benefits and their confidence in making dietary changes to reflect a Mediterranean diet pattern had improved after presentation of the AusMed education materials. Responses are shown in Figure 2.

#### 3.2.2. Phase 1 Program Materials Survey

**Figure 3:**
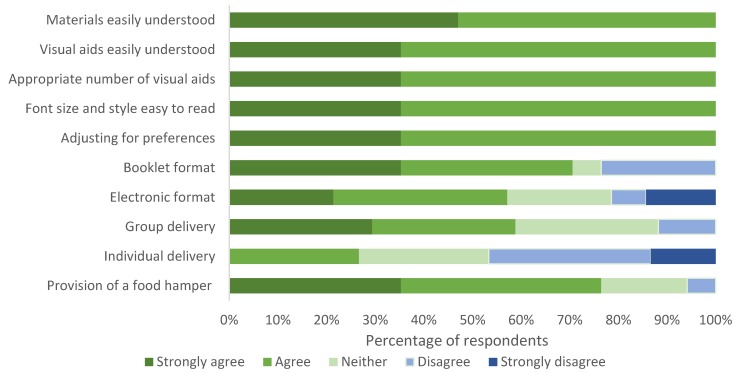
Consumer research group participant (*n* = 17) opinions on the ease of use, effectiveness, applicability and format of AusMed diet program materials.

All participants agreed that the physical materials were easy to understand and visually appealing and that they would be able to use the materials to adjust the meal plan for individual taste preferences while still maintaining the integrity of the program. More participants indicated a preference for a booklet format (70%) rather than an electronic version (57%) and a group setting (strongly agree 29%, agree 29%, disagree 12%, and strongly disagree 0%) rather than individual delivery (strongly agree 27%, agree 0%, disagree 33%, and strongly disagree 13%). The majority of participants agreed that provision of a staple foods hamper would aid in adherence (76%). Responses are shown in Figure 3. 

#### 3.2.3. AusMed Foods Survey Phase 1

**Figure 4:**
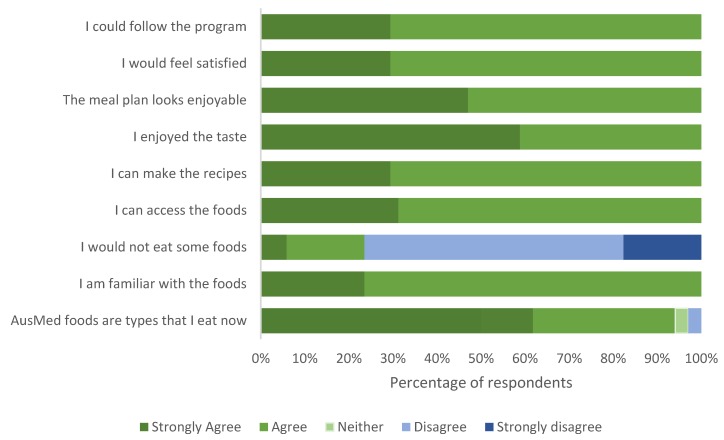
Consumer research group participant (*n* = 17) opinions on the familiarity, accessibility and acceptability of the foods from the AusMed diet program and their ability to adhere to the AusMed meal plan.

Participants completed this survey after a presentation of the recipes, a cooking demonstration and tasting. All participants indicated that they were familiar with foods represented in the AusMed diet program. The majority of participants (89%) reported that their current diet includes some of the foods found in the program and that there were no foods on the program that they would not eat (76%). All participants had confidence that they could access the foods, make the recipes, enjoyed the taste, would feel satisfied by the food and felt confident that they had the ability to follow the program. Participant responses are shown in Figure 4.

### 3.3. Phase 1 Qualitative Analysis

All interviewees had positive feedback for the program materials and found them clear and easy to follow. They also confirmed a willingness to adhere to the AusMed diet program, were confident in their ability to cook the recipes and were willing to experiment with new ingredients. One interviewee did suggest that it was difficult to make a definitive statement of confidence without actually utilising the materials in practice.

*“I guess you don’t really know for sure until you try to follow. It’s the doing of the thing isn’t it, that makes you realise what works and perhaps where the improvements are?” (Participant 6)*.

Detailed thematic analysis of transcribed interviews revealed three common themes—1. barriers to adherence, 2. additional program support and 3. simplification and individualisation of the program materials to improve adherence. Thematic analysis is summarised in Table 2.

### 3.4. Phase 2 Quantitative Analysis

#### 3.4.1. The 14-Point Mediterranean Diet Score

Change in intake can be found in Table 3. Overall, the mean (± SD) baseline Mediterranean Diet MD score was 5.4 ± 2.38. After the 2-week dietary intervention, the mean Mediterranean Diet MD score increased by 4.2, to a total score of 9.6 ± 2.03 (*p* = < 0.001). All mean scores for each individual criterion showed improvement from baseline—except for nuts, which remained the same at 3.53 (±1.96). Post intervention, all participants were using Extra Virgin Olive Oil (EVOO) as their main source of culinary fat and had made a significant increase in their intake, from a mean 1.15 (±0.86) to 2.53 (±1.19) tbsp/day (*p* = 0.001). Significant increases in intake were also found in vegetable (*p* = < 0.001), legume (*p* = < 0.001), fish (*p* = 0.009) and sofrito (*p* = 0.013) intakes. Sofrito is an example of the synergistic effects of cuisine from the Mediterranean dietary pattern and includes ingredients such as olive oil, tomato, onion and garlic, which synergistically enhance the nutritional value of their individual components when combined [20]. Participants also significantly decreased their intake of red meat (*p* = 0.015) and commercial sweets and pastries (*p* = 0.021). All other food groups showed non-significant trends towards an increase in intake.

#### 3.4.2. Phase 2 AusMed Foods Survey 

**Figure 5:**
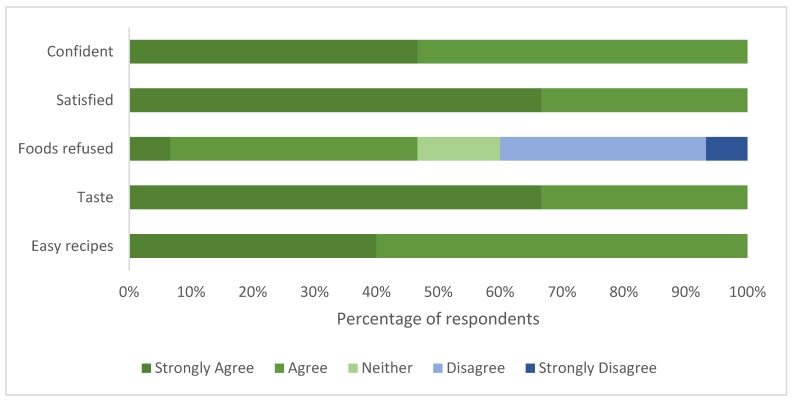
Intervention participant (*n* = 15) opinions on the acceptability and ease of preparation of the foods on the AusMed diet program and confidence in ability to adhere.

All participants reported that they were confident in their ability to maintain the diet program post intervention. They enjoyed the taste of the foods (67% strongly agreed) and felt satisfied for the duration of the intervention (67% also strongly agreed). Responses were varied as to whether there were foods within the program that they would refuse to eat, with 47% agreeing that there were, 13% remaining neutral and 40% disagreeing. Responses are shown in Figure 5.

#### 3.4.3. Phase 2 Text Message Support Evaluation Survey

**Figure 6:**
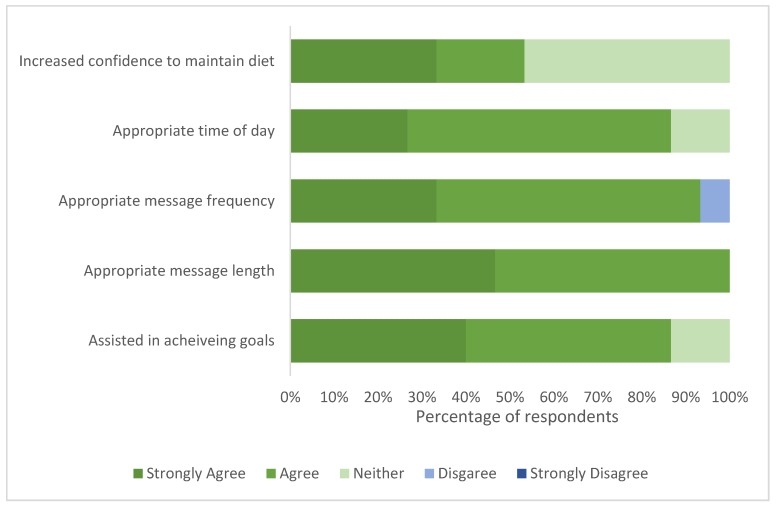
Intervention participant (*n* = 15) opinions on the length, frequency, scheduling and benefit of the text message support component of the AusMed diet program.

The majority of participants agreed that the text messages delivered to support behaviour change and adherence were of an appropriate length (100%), frequency (99%), timing (87%) and were beneficial in achieving their goals (87%). Responses were varied as to whether messages aided confidence for continued adherence—57% agreed and 47% were neutral. Responses are shown in Figure 6.

## 4. Discussion

These results provide preliminary trial evidence of the feasibility of a Mediterranean diet program designed to be specific to an older Australian population. In the Phase 1 process evaluation, participants found that the AusMed diet program materials were easy to use and increased their knowledge and confidence to adhere to a Mediterranean diet pattern. Qualitative analysis identified the complexity of the meal plan, individual food preference and cost to be barriers to adherence, differing from commonly perceived barriers of motivation, time and cost [21]. These differences may be explained by participant demographics (Table 1). The majority of participants were not engaged in full-time work and 85% reported a household income above the highest possible aged pension of $32,210/year [22], meaning time and cost are less likely to be a barrier.

Phase 1 qualitative analysis found that feasibility may be improved with additional support and the simplification/individualisation of program materials. Nutrition education and social support have been identified as strategies that are predictors of adherence to a health behaviour change program for an ageing population [23,24]. Participants identified a group setting and utilising mHealth strategies (the use of mobiles technologies such as phones or tablet devices in health practice) [25] to support nutrition education and behaviour change as preferable methods. 

As a result of data collected in Phase 1, changes were made to the AusMed diet program including the addition of meal plan and shopping list templates and the development of a presentation on their use. This allowed for simplification and individualisation of the program. Interventions that increase the self-efficacy of participants have been shown to be effective in increasing adherence and can be a reliable predictor of behaviour change in an older population [26]. Individual tailoring of dietary interventions and meal plan flexibility has been shown to be a key strategy to improve adherence [27]. A large Mediterranean diet cohort study (*n* = 10,376) showed that similarity between the macronutrient profile of the participants usual diet and the intervention resulted in longer-lasting adherence [28] and it was thought that tailoring an intervention to decrease this gap could improve adherence. The use of menu plan templates allows participants to create a plan similar to their current eating pattern (and macronutrient profile) while still maintaining the nutrient structure of a Mediterranean dietary pattern.

Text message support was also included in the modified program. Participants had a positive response to messages and felt that they aided adherence but had a mixed response in terms of their aid in improving confidence to continue the program. A recent Cochrane review in utilising text message support for preventive health (*n* = 1933) [29] and a meta-analysis of the use of text message support for secondary prevention of cardiovascular disease (*n* = 949) [30] found that while text messages may support preventive health care, evidence is too limited to draw reliable conclusions. A meta-analysis published recently shows promising results in the use of tailored text message support for self-management of type 2 diabetes (*n* = 949), delivering a significant improvement in HbA1C values [g = 0.54 (95% CI: 0.009, 0.99); *p* = < 0.0001)] [31]. Tailoring of the text message support using individual learning goals and the COM-B framework may improve its efficacy in subsequent trials.

In Phase 2 pilot testing of the AusMed diet program, a population of older Australians were able to improve their adherence to a Mediterranean style diet. An adherence of ≥ 10 is considered ‘excellent’ [18,32]. Participants’ mean score improved from a baseline of 5.4 (±2.32), which is considered to be ‘fair’ adherence, to a post intervention score of 9.4 (±2.03), which is considered ‘good’ adherence [18,32]. While trial adherence was not considered to be ‘excellent’, the comparative scores were validated in a Spanish population and, as such, may have had a closer adherence to a Mediterranean dietary pattern at baseline [32]. It is important to note that two participants in the Phase 2 trial had not participated in Phase 1 and, as such, may have had a different understanding of the AusMed which may have influenced results.

Participants reported a significant increase in the consumption of foods that have been shown to improve CVD risk, EVOO (relative risk 0.83, 95% CI 0.77, 0.89), legumes (RR: 0.90; 95% CI: 0.83, 0.98) and vegetables (RR: 0.87; 95% CI: 0.77, 0.98) [33], and a significant reduction in foods that have been shown to increase CVD risk, red meat (RR: 0.97; 95% CI: 0.88, 1.07) and commercial sweets [34,35]. A longer trial with increased behaviour change support is needed to show whether adequate intake can be achieved in the long term in order to see health benefits.

Participants’ responses to the foods from the AusMed diet program were generally positive. All enjoyed the taste, found the recipes easy to make and were satisfied by the foods in the program. The majority reported increased self-efficacy and confidence in their ability to maintain adherence post intervention. Palatability and satisfaction are linked with higher compliance to a Mediterranean diet pattern [8,13].

There is merit to using a mixed-methods approach to evaluate dietary program feasibility in order to improve design prior to a larger intervention trial to increase adherence. Quantitative survey and adherence data give baseline statistics and a measure of participant confidence, while the qualitative analysis provides in-depth data on the individual opinion of each facet of the program and potential barriers to acceptability. This provides the opportunity to create program materials that address those barriers to increase adherence in a larger trial. The use of a retirement community for recruitment proved effective and further research of this as a method of increasing engagement with health behaviour change programs may beneficial.

There are, however, limitations associated with the data in this report. The population was small, which limits statistical power (*n* = 17 in Phase 1 and *n* = 15 in Phase 2), and may not be representative of older Australians in general. The sample was a ‘well’ population of higher socioeconomic status and education level and, as such, may not be generalized to older Australians. At baseline, they reported a high level of confidence in their ability to access and prepare food. They were also motivated to attend a 2–3 h presentation away from home—all of which may not be indicative of the predicted adherence of an average older Australian population. Intervention intake data were self-reported and, as such, may be subject to bias. A Food Frequency Questionnaire (FFQ) or 24 h recall would give a more detailed and robust description of adherence. A trial length of 2 weeks is not indicative of long-term adherence and there was no control group. While we collected baseline data, there is inherent bias in the lack of a control group for comparison.

## 5. Conclusions 

In this study, a population of older Australian’s were found to improve their adherence to a Mediterranean dietary pattern modified to be specific to the Australian context. Data collected were suggestive of the feasibility of the AusMed diet program and this preliminary data will be used to inform a future intervention in the efficacy of a Mediterranean diet program modified for a population of Australian stroke survivors utilising mHealth. Larger randomised controlled trials conducted over a longer period with a sample from across a broader range of older Australians (including those from various socioeconomic and cultural backgrounds) are needed to confirm whether the AusMed diet program can increase adherence to a Mediterranean dietary pattern and whether there are associated health benefits for older Australians. 

## Figures and Tables

**Table 1:** Participant characteristics.

Variables	All Subjects (*n* = 17)	Female (*n* = 12)	Male (*n* = 5)
Age (years)	71.2 ± 4.2	70.7 ± 4.2	72.8 ± 4.2
Marital status			
Married	13 (76%)	8 (67%)	5 (100%)
Divorced	2 (12%)	2 (17%)	-
Single	1 (6%)	1 (8%)	-
Widowed	1 (6%)	1 (8%)	-
Education			
School certificate/HSC	7 (41%)	6 (50%)	1 (20%)
Certificate/diploma	6 (35%)	4 (33%)	2 (40%)
University degree	4 (24%)	2 (17%)	2 (40%)
Household income			
Rather not say/unknown	3 (17%)	3 (25%)	-
$25,000 to $49,999	9 (53%)	7 (58%)	2 (40%)
$50,000 to $99,999	3 (17%)	2 (17%)	1 (20%)
$100,000 to $199,000	2 (13%)	-	2 (40%)

Additional participant data from Phase 2 (*n* = 2) were: age, 64.5; female, 100%; married, 50%; divorced, 50%; education—school certificate/HSC, 50%; university degree, 50%; household income—$50,000 to $99,999, 50%; $100,000 to $199,000, 50%. Phase 2 Participant characteristics (*n* = 15) in Supplementary Table S2.

**Table 2:** Thematic analysis of semi-structured interviews (*n* = 6) from Phase 1—the acceptability and feasibility of materials from the AusMed diet program.

Theme	Sub-Theme	Verbatim Evidence
Barriers to adherence	Food preference—flexibility of menu to allow for individual preference Complexity and size of meal plan Perceived additional cost	*“I think it’s overcoming the fat issue, you know, the oils and the…even though I use the olive oil and sprays, it’s the amount” Participant 5* *“The meal plan is very big. I know you said in the presentation that it is flexible but there is a lot of it. I tend to have the same thing for breakfast most days and sometimes a fairly similar type thing for lunch so the, just the sheer number of recipes is a little overwhelming for me” Participant 2* *“But it’s when you first look at it, I thought, Oh goodness me! And then I thought, no, come on, you’ve probably got just about everything that’s on there already in the house. That’s just my first impression of the list” Participant 5* *“What I’d like to do is eat more fish, I went fishing and we caught some lovely whiting, so it will be easier, but it is expensive. I love sardines and mackerel and all those sorts. We do get a bit of canned stuff, that works for us” Participant 4*
Additional support	Group support—several found group support preferable but one participant was firmly against mHealth—website, application and text messaging to reinforce behaviour change	*“We can form a Mediterranean club here…like some people play cards, some go bowling, some go fishing and so on…. not just ourselves, but encourage other people to come into the group as well” Participant 1* *“No, no, I wouldn’t go. No, no, no, not a group. No, I don’t think it’s, well for me, it’s not the environment that I would have.” Participant 2* *“I’ll be more than happy with an app or something like that…. if you’re away from home for quite a period of time and still wanting to follow the guidelines then, to have an app to refer to…” Participant 5* *“Well yes, absolutely yes! If you did it, that text message then that would be fun. Absolutely yes. A text message would be my preferred method to get information” Participant 2*
Simplification and individualisation of materials	‘Food hacks’ Addition of ‘how to season without salt’ advice Templates to allow participants to build individual plans Single meal plan	*“Well, I was even thinking of some of those recipes I’d cheat with, the chicken and leek pot pies, we’d buy a chicken already done…a few little cheats or hacks for other people” Participant 2* *“I mean, you know, we know salt in moderation, but instead of, maybe some tips on how to flavour and season your food” Participant 5* *“…a sort of template thing…where you pick and choose your ingredients...where you tick boxes, perhaps a meal template or a plan template to make our own plan” Participant 6* *“I’m on my own too, so I think there might be just too much. I tend to keep things pretty simple” Participant 5*

**Table 3:** Mean change in intake of 14-point MDS baseline to post intervention.

MD Score Individual Questions	Baseline (*n* = 15) Mean ± SD	Post Intervention (*n* = 15) Mean ± SD	*p*
Total MD Score	5.4 ± 2.38	9.6 ± 2.03	<0.001
1. Use EVOO as main culinary fat	73%	100%	* 0.099
2. Olive oil tbsp/day	1.15 ± 0.86	2.53 ± 1.19	0.001
3. Vegetable serves/day	1.20 ± 0.47	1.71 ± 0.59	<0.001
4. Fruit serve/day	1.83 ± 0.59	2.20 ± 0.94	0.135
5. Red meat serves/day	1.13 ± 0.71	0.65 ± 0.63	0.015
6. Butter/cream/margarine serves/day	0.82 ± 1.05	0.50 ± 0.49	0.237
7. Soft drink serves/day	0.08 ± 0.26	0.07 ± 0.26	0.237
8. Wine glasses/week	10.27 ± 7.04	10.67 ± 6.15	0.620
9. Legumes serves/week	0.47 ± 0.64	1.8 ± 0.94	<0.001
10. Fish or shellfish serves/week	3.20 ± 2.57	4.33 ± 2.32	0.009
11. Commercial sweets/pastries serves/week	1.60 ± 1.72	0.67 ± 0.82	0.021
12. Nuts serves/week	3.53 ± 3.83	3.53 ± 1.96	1.000
13. White meat preferentially	80%	87%	* 1.000
14. Sofrito serves/week	0.93 ± 0.59	2.07 ± 1.62	0.013

*p*-Values corresponding to paired t-tests, *p*-Values * corresponding to Fisher’s Exact test, *p* < 0.05 = significant, MD = Mediterranean Diet, EVOO = Extra Virgin Olive Oil, and sofrito = braised combination of EVOO, garlic, onion and tomato.

## Supplementary Materials

The following are available online at https://www.mdpi.com/2072-6643/12/4/1044/s1, Table S1: Text messages and COM-B model for behaviour change. Table S2: Phase 2 participant characteristics. Figure S1. Participants (*n* = 17) Consumer research group opinions on program food palatability and confidence in food access and preparation skill.
