# Supplementary material for: Feasibility of the AusMed Diet Program: Translating the Mediterranean Diet for Older Australians
Source: Nutrients. 2020 Apr 10;12(4):1044. doi: 10.3390/nu12041044 (PMC7230642; doi:10.3390/nu12041044)
Supplement: Supplementary file 1 [file nutrients-12-01044-s001.pdf]

Table S1. Text messages and COM-B model for behaviour change.

COM-B	TDF	What needs addressing	Intervention function	Behaviour change technique
**1. Capability - psychological**	Knowledge	Lack of knowledge of nutrient make up of Mediterranean eating pattern	Education	Comparative imaging of future outcomes
**TEXT 1: "Make vegetables the hero of your dish (even at breakfast!) – remember one big difference between the Australian and Mediterranean diet patterns is the ratio of plant to animal foods. Making vegies the star of your plate helps maintain your health for longer"**				
**2. Capability - physical**	Knowledge	Lack of knowledge around personalising the menu plan for increased uptake	Enablement<br>Education	Instruction of how to perform behaviour<br>Prompts and cues
**TEXT 2: "Don't forget to personalise your shopping list by adding in your choice of salads and taking into account any other changes you've made to make it your own – if you own it, you're more likely to eat it, enjoy it and stick to it!"**				
**3. Opportunity - physical**	Environment, context and resources	The perception that time is a barrier to changing dietary patterns	Enablement	Instructions on how to perform behaviour<br>Prompts and cues
**TEXT 3: "A tip for the time poor, prep like a chef! Have takeaway type containers in your fridge/pantry with the makings of a meal all ready to go. Wash and prepare salad leaves, roast root vegetables, toast nuts and store them in airtight containers, poach some chicken, grill some lamb – and have it all there ready there to grab and go."**				
**4. Motivation - reflective**	Belief about capabilities<br>Optimism	Affirming the importance of goals setting and monitoring	Persuasion<br>Education	Framing/reframing<br>Decreasing negative emotions
**TEXT 4: "Time for reflection – take some time to think about the goals you set for yourself in our session – are your steps still heading toward them or do you need to change direction? Just remember there is more than one way to get to your destination. Take note of what's working for you and change what's working against you. You can do it!"**				
**5. Motivation - automatic**	Emotion	Fear of failure	Education<br>Persuasion	Reducing negative consequences<br>Framing/reframing
**TEXT 5: "As we approach the end of our 2-weeks remember that it is your overall eating pattern that is important. Eat a rainbow, eat variety and remember – if you have a meal that doesn't fit the pattern it's not the end of the world, it's what your diet looks like overall that will have the long-lasting benefit for your health"**				

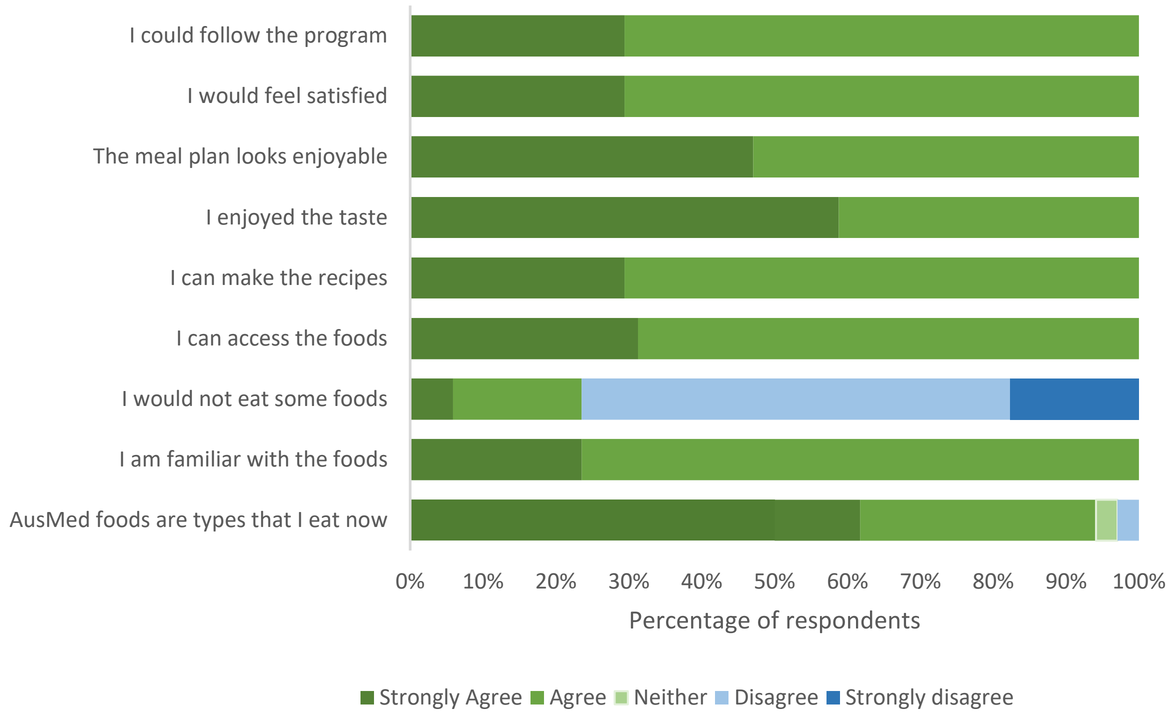

Figure S1. Participants (n=17) Consumer research group opinions on program food palatability and confidence in food access and preparation skill.

Table S2. Phase 2 Participant characteristics

Variables	All subjects (n=15)	Female (n=12)	Male (n=3)
Age (years)	70.4 ± 6.1	69.6 ± 6.1	73 ± 5.1
Marital status			
Married	11 (73%)	8 (67%)	3 (100%)
Divorced	1 (12%)	1 (17%)	—
Single	1 (6%)	1 (8%)	—
Widowed	1 (6%)	1 (8%)	—
Education			
School certificate/HSC	6 (40%)	5 (42%)	1 (33%)
Certificate/diploma	5 (33%)	5 (42%)	—
University degree	2 (13%)	2 (16%)	2 (66%)
Household income			
Rather not say/unknown	5 (33%)	5 (42%)	—
$25,000 to $49,999	6 (40%)	5 (42%)	1 (33%)
$50,000 to $99,999	3 (20%)	2 (16%)	1 (33%)
$100,000 to $199,000	1 (7%)	—	1 (33%)
